# Clinical Relevance of Gain-Of-Function Mutations of p53 in High-Grade Serous Ovarian Carcinoma

**DOI:** 10.1371/journal.pone.0072609

**Published:** 2013-08-13

**Authors:** Hyo Jeong Kang, Sung-Min Chun, Kyu-Rae Kim, Insuk Sohn, Chang Ohk Sung

**Affiliations:** 1 Department of Pathology, Asan Medical Center, University of Ulsan College of Medicine, Seoul, Korea; 2 ASAN Center for Cancer Genome Discovery, Asan Medical Center, University of Ulsan College of Medicine, Seoul, Korea; 3 Samsung Cancer Research Institute, Seoul, Korea; University of Quebec at Trois-Rivieres, Canada

## Abstract

**Purpose:**

Inactivation of *TP53*, which occurs predominantly by missense mutations in exons 4–9, is a major genetic alteration in a subset of human cancer. In spite of growing evidence that gain-of-function (GOF) mutations of p53 also have oncogenic activity, little is known about the clinical relevance of these mutations.

**Methods:**

The clinicopathological features of high-grade serous ovarian carcinoma (HGS-OvCa) patients with GOF p53 mutations were evaluated according to a comprehensive somatic mutation profile comprised of whole exome sequencing, mRNA expression, and protein expression profiles obtained from the Cancer Genome Atlas (TCGA).

**Results:**

Patients with a mutant p53 protein (mutp53) with a GOF mutation showed higher p53 mRNA and protein expression levels than patients with p53 mutation with no evidence of GOF (NE-GOF). GOF mutations were more likely to occur within mutational hotspots, and at CpG sites, and resulted in mutp53 with higher functional severity (FS) scores. Clinically, patients with GOF mutations showed a higher frequency of platinum resistance (22/58, 37.9%) than patients with NE-GOF mutations (12/56, 21.4%) (*p*=0.054). Furthermore, patients with GOF mutations were more likely to develop distant metastasis (36/55, 65.5%) than local recurrence (19/55, 34.5%), whereas patients with NE-GOF mutations showed a higher frequency of locoregional recurrence (26/47, 55.3%) than distant metastasis (21/47, 44.7%) (*p*=0.035). There were no differences in overall or progression-free survival between patients with GOF or NE-GOF mutp53.

**Conclusion:**

This study demonstrates that patient with GOF mutp53 is characterized by a greater likelihood of platinum treatment resistance and distant metastatic properties in HGS-OvCa.

## Introduction

Loss of p53 function is a common feature in human cancers [1] and mutation is a major cause of loss of p53 function in a subset of tumor. *TP53* mutation is a major cause in almost every type of human cancer [1]. While other tumor suppressors, such as *RB, APC*, or *BRCA1*, are commonly inactivated by frame-shift or nonsense mutations, missense mutation is predominant type in *TP53* mutation in human tumors. These mutations occur primarily in exons 4–9, which encode the DNA-binding domain of the protein [2,3]. Single base substitutions frequently occur at CpG dinucleotide sites, resulting in C (cytosine) : G (guanine) > T (thymine) : A (adenine). Therefore, a tumor cell with a *TP53* missense mutation produces full-length p53 protein with only a single amino acid substitution. Mutant p53 protein (mutp53) resulting from missense mutations has prolonged half-life and accumulate within the tumor cells. Mutp53 may have properties that can contribute to tumor progression. Many mutant forms of p53 can bind and inactivate p53-related proteins such as p63 and p73 [4]. Functionally, this additional oncogenic activity of mutp53 has been described as gain-of-function (GOF), which was demonstrated to drive tumor cells toward migration, invasion, and metastasis in mouse models [5–10]. However, little is known about the clinical relevance of GOF mutp53, and there is so far no concrete evidence that GOF mutations in p53 contribute to clinical behavior in human cancers.

There are several hotspot mutation sites in p53, such as R175, G245, R248, R249, R273, and R282, which are frequently mutated in cancer [3,11]. These sites are thought to be hotspots for mutations because of the susceptibility of particular codons to carcinogen-induced alterations, and because mutations at these sites give the mutated cells growth and survival advantages [3]. GOF may also play a significant role in the positive selection of missense mutations in *TP53* during tumorigenesis. However, still, there is no definitive evidence on the relationship between GOF and selection of mutp53 in human cancers.

Many studies on GOF mutp53 have been performed; however, no systematic study using a large number of samples investigated the clinical relevance of GOF properties in mutp53. High-grade serous ovarian carcinoma (HGS-OvCa) has been reported to have a high percentage of *TP53* mutations [12,13]. A recent study using high-throughput sequencing technology demonstrated that *TP53* mutations occurred in 96% of 316 HGS-OvCa samples [14]. Therefore, a study using this cohort can give insight into the clinical and pathological features of HGS-OvCa tumors with GOF mutp53. In this study, we evaluated the clinicopathological features of tumors with hotspot or GOF mutations of *TP53* using a comprehensive somatic mutation profile comprised of whole exome sequencing, mRNA expression profiles, and protein expression profiles obtained from The Cancer Genome Atlas (TCGA).

## Materials and Methods

### Somatic mutation, mRNA, and protein expression data

Whole exome sequencing data from 301 patients with HGS-OvCa who had *TP53* mutations were downloaded from the open-access TCGA website (http://tcga-data.nci.nih.gov) [14]. Data were downloaded on October 29, 2011. The sequencing, quality control, and validation procedures are described elsewhere [14]. *TP53* mRNA expression data were obtained from unified expression microarray data for all 301 patients [14]. Procedures, platforms used, normalization, and processing methods for expression microarray data have been described [14]. Normalized protein expression data from a reverse-phase protein array (RPPA) (a high-throughput antibody-based technique) composed of 165 proteins were downloaded from the TCGA website. The detailed procedures, proteins evaluated, processing, and analysis methods were described in previous reports [15,16]. Protein expression data were available for 196 of the 301 patients. Clinical information, including age, overall survival, progression-free survival, platinum response (sensitive or resistant), recurrence type (locoregional or distant metastasis), tumor grade, and tumor stage were obtained from all patients. These parameters are defined in previous reports [14,17,18].

### Measurement of functional severity of mutp53

To predict the consequences of missense mutations resulting in mutp53, functional severity (FS) scores for each of the 92 mutant proteins from 185 patients with missense mutations were calculated from Web Serve (http://www.ifm.liu.se/bioinfo) using the PREDMUT algorithm developed by Carlsson et al. [19]. Briefly, the algorithm used 12 parameters, including accessibility, similarity of the surroundings, DNA/zinc, Pocket/cavity, calculated energy, average calculated energy, secondary structure, hydrophobicity difference, size difference, amino acid similarity, polarity change, and conservation. Detailed descriptions of each parameter have been described [19].

### Calculation of GC content and CpG density

The *TP53* cDNA normal reference sequence was downloaded from COSMIC (Catalogue Of Somatic Mutations In Cancer) (http://www.sanger.ac.uk/). From the *TP53* cDNA, the GC content per 50 base pairs was calculated as follows: [(G or C)/(A+T+C+G)] × 100. The CpG density per 50 base pairs was also calculated, as follows: (number of CpG dinucleotides per 50 base pairs/25) × 100.

### Hotspot mutation

A site was considered a hotspot if mutations were detected in five or more of the 301 patients. Hotspot mutations were evaluated separately based on cDNA and on protein sequences, because different form of mutp53 can result from mutations at the same cDNA site; for example, mutations at c818 resulted in three different mutant proteins, R273L (n=2), R273H (n=11), and R273P (n=1). Therefore, c818 was considered a mutation hotspot at the cDNA level, while of the resulting proteins, only R273H qualified as a hotspot mutation. Hotspot mutations at the protein level were evaluated to assess the FS of mutp53 proteins and to correlate their severity with the patients’ clinicopathological features.

### Classification of GOF activity in mutp53

Using three categories of GOF activity, including 1) interference with p73 activity, 2) transactivation of genes repressed by wild-type p53, and 3) cooperation with oncogenes for transformation of rat embryonic fibroblast or mouse embryonic fibroblast cells, 103 mutp53 proteins were evaluated [20]. In this study, a mutp53 was classified as having a GOF mutation when it satisfied at least one of the three criteria. According to these criteria, 31 mutp53 forms (S127Y, P151S, R156P, Y163N, Y163C, V173L, R175H, C176Y, H179R, H179Q, L194R, Y205C, H214R, Y220C, Y234C, M237I, S241F, G245C, G245S, G245V, G245D, R248W, R248G, R248Q, R273C, R273L, R273H, R273P, C275Y, D281G, and R282W) were classified as GOF mutations. The remaining mutp53 forms were classified as having no evidence of GOF activity (NE-GOF). In total, 101 of 187 patients with missense mutations were considered to have GOF mutp53.

### Differential protein expression between GOF and NE-GOF in patients

Differential protein expression analyses comparing GOF and NE-GOF mutp53 were performed in the 126 patients in whom protein expression data were available using BRB-Array Tools (version 4.2.1 stable release), which was developed by Dr. Richard Simon and the BRB-Array Tools Development Team [21]. To analyze differential protein expression between the two groups, parametric p-values, permutated p-values based on 10000 random permutations, and the false discovery rate (FDR) were calculated. Volcano plot was used to demonstrate the differentially expressed proteins. Briefly, the differences (log2fold change) between the two groups were plotted on X-axis and the -log_10_ (*p* value) was plotted on the Y-axis. In addition, the significance analysis of microarrays (SAM) method [22], which was another method can be used to pick out significant genes based on differential expression between groups, was also applied to identify differentially expressed proteins using a target FDR of 0.25, 10000 permutations, and an exchangeability factor of 90. Interactive plot of the observed versus expected (based on the permuted data) d-values was generated by SAM method.

### Statistical Analysis

T-test or Wilcoxon rank sum test was used to evaluate differences in means for continuous data between the two groups. The Chi-squared test or the Fisher’s exact test was used to test the association between the two categorical groups. Overall survival and progression-free survival were determined using the Kaplan-Meier method, and survival curves were compared using the log-rank test. All tests were two-sided and *p*-values less than 0.05 were considered statistically significant. Statistical analysis was performed using Stata/IC statistical software (version 12, StataCorp Ltd., TX) and the R program (version 2.14.0: www.r-project.org).

## Results

### The relationship between GC content, CpG density, and hotspot mutations

**Figure 1:**
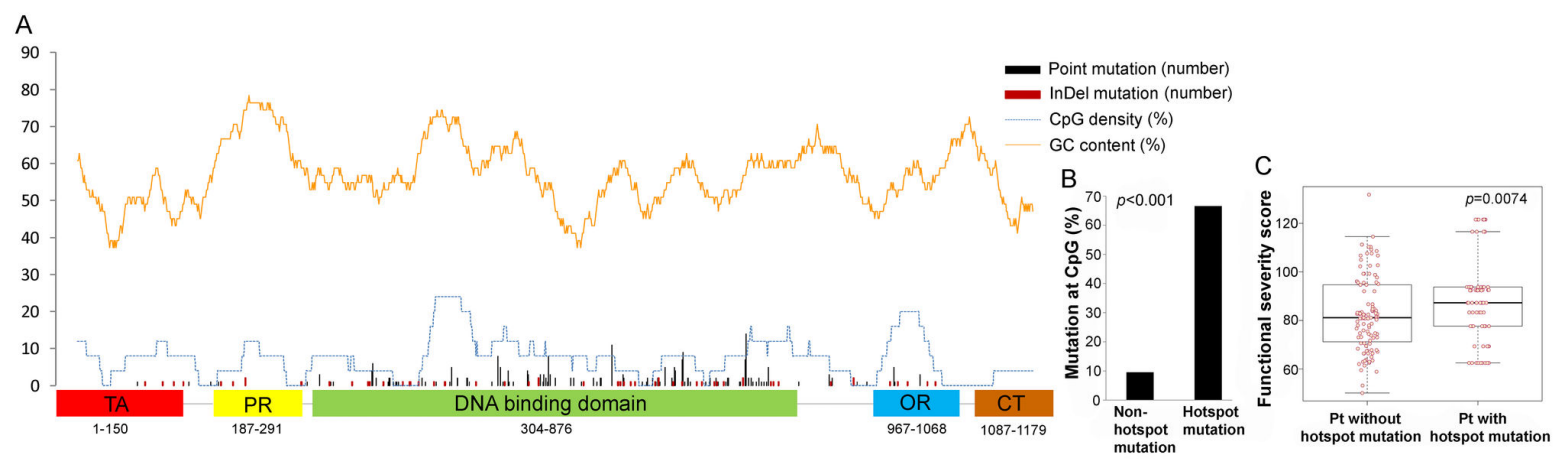
Guanine and cytosine (GC) content, CpG site, and hotspot mutations in *TP53*. Frequencies of *TP53* mutations and their locations with GC content and CpG density. Most mutations were located in DNA binding domain (A). Hotspot mutations (five or more frequencies in 301 patients) were more likely to occur at CpG sites (*p*<0.001, chi-square test) (B). Functional severity scores of hotspot mutant p53 proteins (n=74) were much higher than those of p53 mutant proteins that occurred outside of the hotspots (n=111) (*p*=0.0074, Wilcoxon rank sum test) (C).

There were 12 hotspot mutation sites at the cDNA level, at positions c469, c524, c527, c584, c659, c722, c733, c742, c743, c817, c818, and c844. Hotspot mutations were located only in DNA-binding domains and all were missense mutations. Generally, hotspot mutations were located in the area of *TP53* cDNA with the highest GC content (Figure 1A), and hotspot mutations were significantly associated with CpG sites (*p*<0.001, Figure 1B). Of a total of 96 single base substitution mutations, 8 of 12 hotspot mutations (66.6%) occurred at a CpG site. However, only a few hotspot mutation sites were present, even though *TP53* cDNA contains broad CpG-dense regions. For example, the proline-rich domain and oligomerization domain of *TP53* have no hotspot mutation sites, despite their high CpG density and GC content. This suggests that hotspot mutations occur selectively within sites that increase the carcinogenic potential of mutp53. Indel mutations occurred throughout the entire *TP53* cDNA, regardless of GC content and CpG density. At the protein level, frequently occurring mutations (≥5 occurrences) were V157H, R175H, C176Y, I195T, Y220C, R248W, R248Q, R273C, R273H, and R282W. Patients with these mutp53 had higher FS scores than patients with p53 mutations outside of hotspots (*p*=0.0074, Figure 1C).

### Expression of p53 mRNA and protein in mutp53

**Figure 2:**
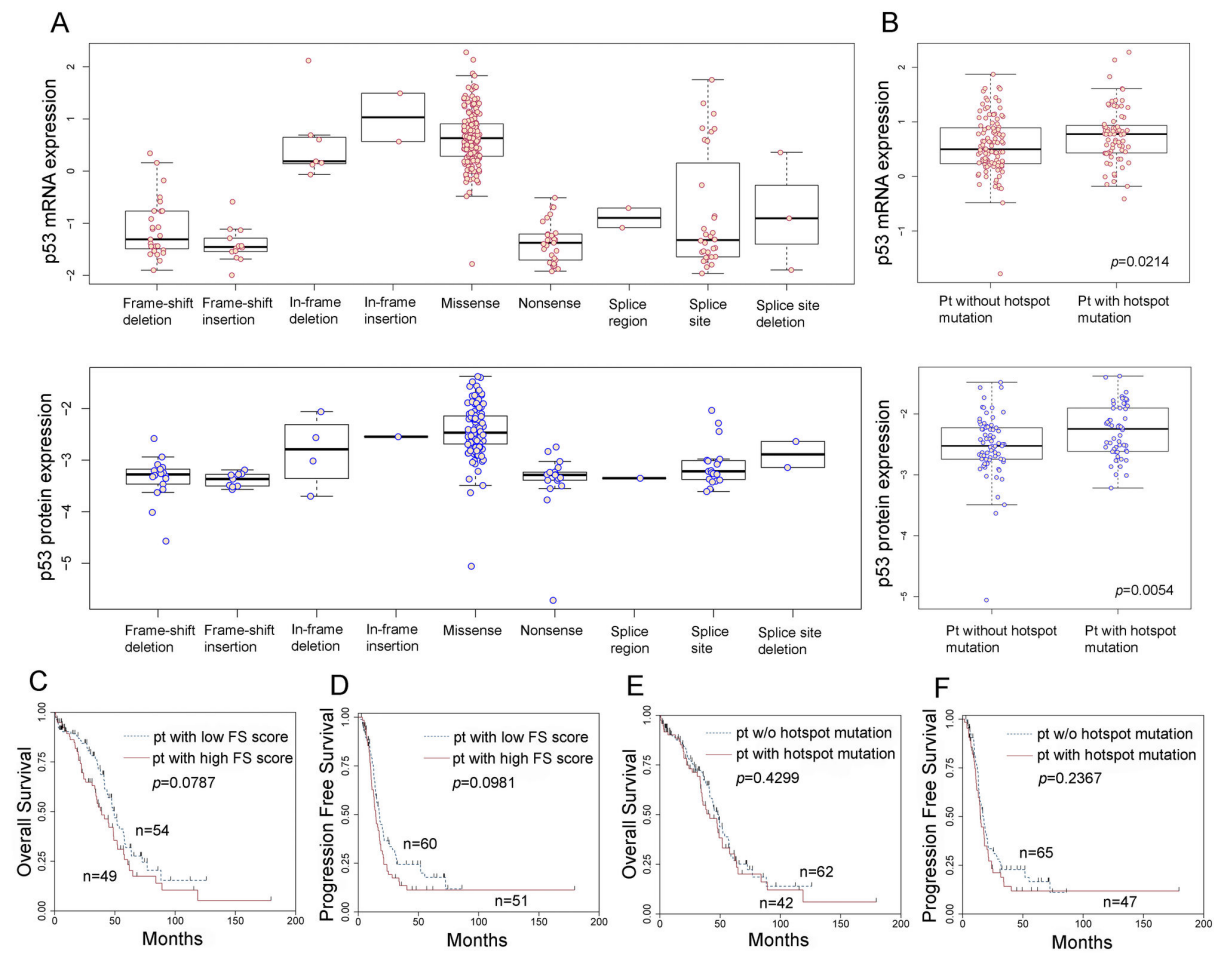
Expression of p53 mRNA, protein, mutation type, and survival in high grade serous ovarian carcinoma. Expression levels of p53 mRNA and protein according to mutation type were present. Higher level of expression was present in in-frame deletion, in-frame insertion, and missense mutation (A). Higher expressions of p53 mRNA (*p*=0.0214, t-test) and protein (*p*=0.0054, t-test) were present in tumors with hotspot mutation (n=72 and n=54, respectively) than mutation in non-hotspot (n=113 and n=73, respectively) (B). In overall (C) and progression-free survival (D) according to functional severity (FS) scores for mutant p53 proteins in patients (pt) with high-grade serous ovarian carcinoma, pt with high FS score showed poorer prognosis but statistically not significant. In overall (E) and progression-free survival (F) according to mutation type, there were no survival differences. Log-rank test was used for survival analysis.

According to mutation type, missense mutations, in-frame deletions, and in-frame insertion mutations had the highest *TP53* mRNA expression levels (Figure 2A). Other mutation types, including nonsense mutation, frame-shift deletion, frame-shift insertion, and slice-related mutation, showed lower levels of *TP53* mRNA expression. Protein expression corresponded to the mRNA expression, and was highest in patients with missense mutations, in-frame deletions, and in-frame insertion mutations. Patients with missense mutations within mutp53 hotspots had higher mRNA expression than patients with mutations outside of p53 hotspots (0.75±0.509 vs. 0.56±0.525, *p*=0.0214, Figure 2B). The p53 protein expression level was also higher in patients with p53 mutations within hotspots than in patients with p53 mutations outside hotspots (-2.27±0.428 vs. -2.52±0.510, *p*=0.0054).

### Functional severity and survival impact of mutp53

In patients with missense mutations, patients with high FS scores for mutp53 tended to show poorer overall (Figure 2C) and progression-free survival (Figure 2D) than patients with low FS scores, although statistical significance was not reached for either overall (*p*=0.0787) or progression-free survival (*p*=0.0981). When patients were divided into two groups according to whether the p53 mutation occurred within or outside of a mutational hotspot, there was no significant difference in either overall (*p*=0.4299, Figure 2E) or progression-free (*p* = 02367, Figure 2F) survival.

### Characteristics of mutp53 with gain-of-function activity

**Figure 3:**
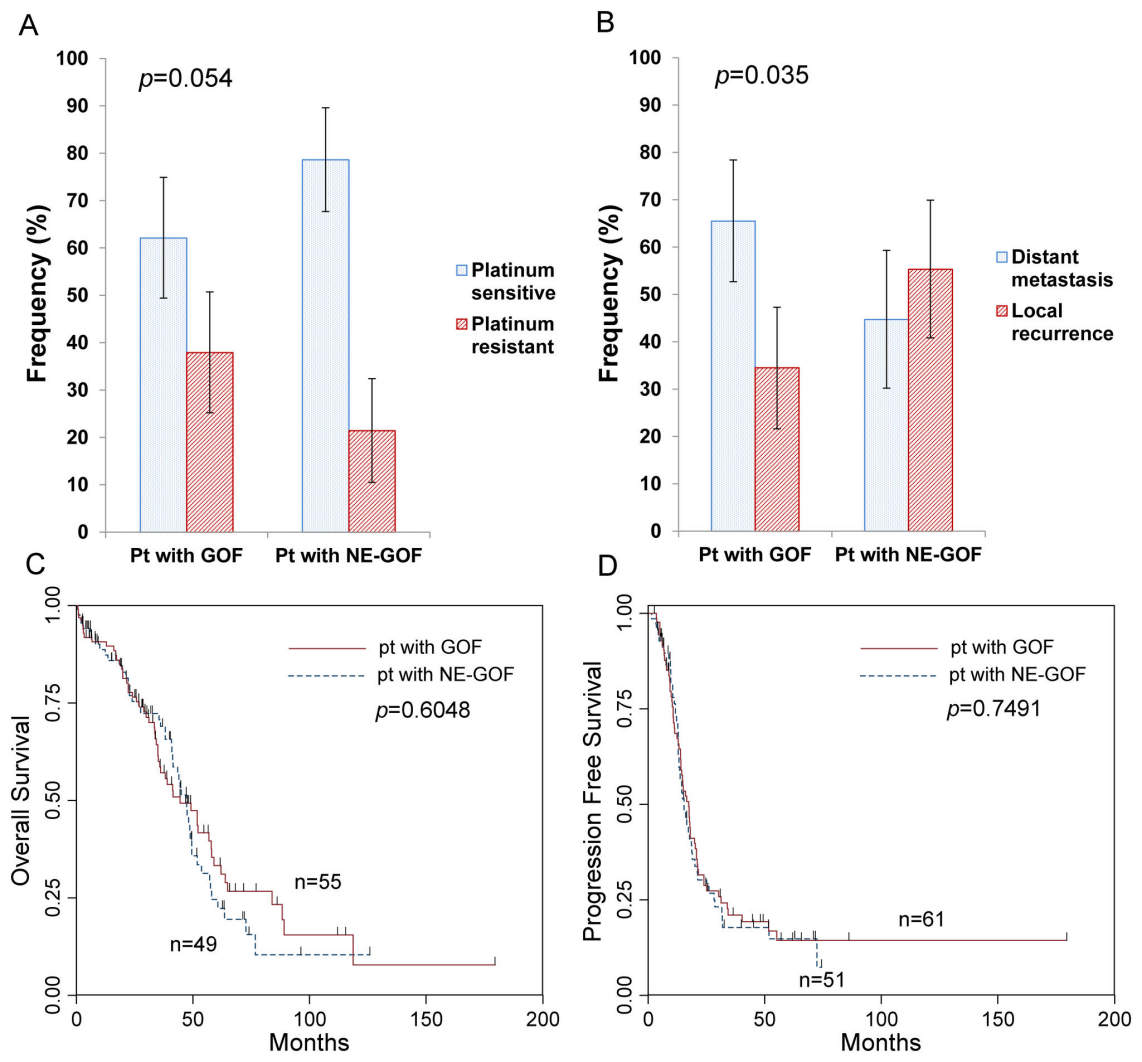
Clinical features and gain-of-function (GOF) mutations in p53. Patients with GOF mutations showed a higher frequency of platinum resistance (22/58, 37.9%) than patients with no evidence (NE)-GOF mutations (12/56, 21.4%) (*p*=0.054, chi-square test) (A). Patients with GOF mutations showed a higher frequency of distant metastasis (36/55, 65.5%) than patients with NE-GOF (21/47, 44.7%) (*p*=0.035, chi-square test) (B). No differences in overall (C) and progression free survival (D) between patients with GOF and NE-GOF mutations were identified. Log-rank test was used for survival analysis.

Patients with GOF mutations showed higher mRNA (*p*=0.0321) and protein (*p*=0.0129) expression than patients with NE-GOF mutations. However, MDM2 mRNA expression level was not different between GOF and NE-GOF (*p*=0.4365) (Table 1). GOF mutations were more likely to result from hotspots mutations (*p*=0.002) and mutations within CpG sites (*p*<0.001), and had higher FS scores than NE-GOF mutations (*p*<0.001). Clinically, patients with GOF mutations showed a higher frequency of platinum resistance (22/58, 37.9%) than patients with NE-GOF mutations (12/56, 21.4%), and a lower frequency of platinum sensitivity (36/58, 62.1%) than patients with NE-GOF mutations (44/56, 78.6%), although this was marginally significant (*p*=0.054) (Figure 3A). Furthermore, there was a different recurrence pattern between patients with GOF mutp53 and patients with NE-GOF mutp53. GOF mutations were associated with the development of distant metastasis (36/55, 65.5%) rather than local recurrence (19/55, 34.5%), whereas patients with NE-GOF mutations showed a higher frequency of locoregional recurrence (26/47, 55.3%) than distant metastasis (21/47, 44.7%) (*p*=0.035, Figure 3B). However, there were no differences in overall (*p*=0.6048, Figure 3C) or progression-free survival (*p*=0.7491, Figure 3D) between patients with GOF and NE-GOF mutations. Other clinicopathological features, including age, grade, and stage were not different between the two groups (Table 1).

**Table 1:** Characteristic of mutant p53 protein with gain of function in high grade serous ovarian carcinoma.

Parameter	Total no.	NE-GOF	GOF	*p* value
Age, yr (median)	179	60.6	60.6	0.9983
Grade				
G2	16	9 (56.3%)	7 (43.8%)	0.436
G3	165	76 (46.1%)	89 (53.9%)	
Stage				0.512
2	7	2 (28.6%)	5 (71.4%)	
3	142	69 (48.6%)	73 (51.4%)	
4	36	15 (41.7%)	21 (58.3%)	
p53 mRNA level, log_2_ ratio	185	0.55 ± 0.55	0.72 ± 0.48	0.0321
p53 protein level, log_2_ ratio	127	-2.54 ± 0.54	-2.32 ± 0.421	0.0129
MDM2 mRNA level, log_2_ ratio	185	-0.07 ± 0.46	-0.13 ± 0.52	0.4365
Hotspot mutation				
no	84	61 (72.6%)	23 (27.4%)	0.002
yes	10	2 (20.0%)	8 (80.0%)	
Mutation at CpG site				
no	77	58 (75.3%)	19 (24.7%)	<0.001
yes	17	5 (29.4%)	12 (70.6%)	
FS score for mutp53	185	79 ± 14.9	89.8 ± 15.5	<0.001

NE-GOF, no evidence of gain-of-function; FS, functional severity; ±, standard deviation; mutp53, mutant p53 protein

### Differential protein expression between patients with GOF and NE-GOF mutations

**Figure 4:**
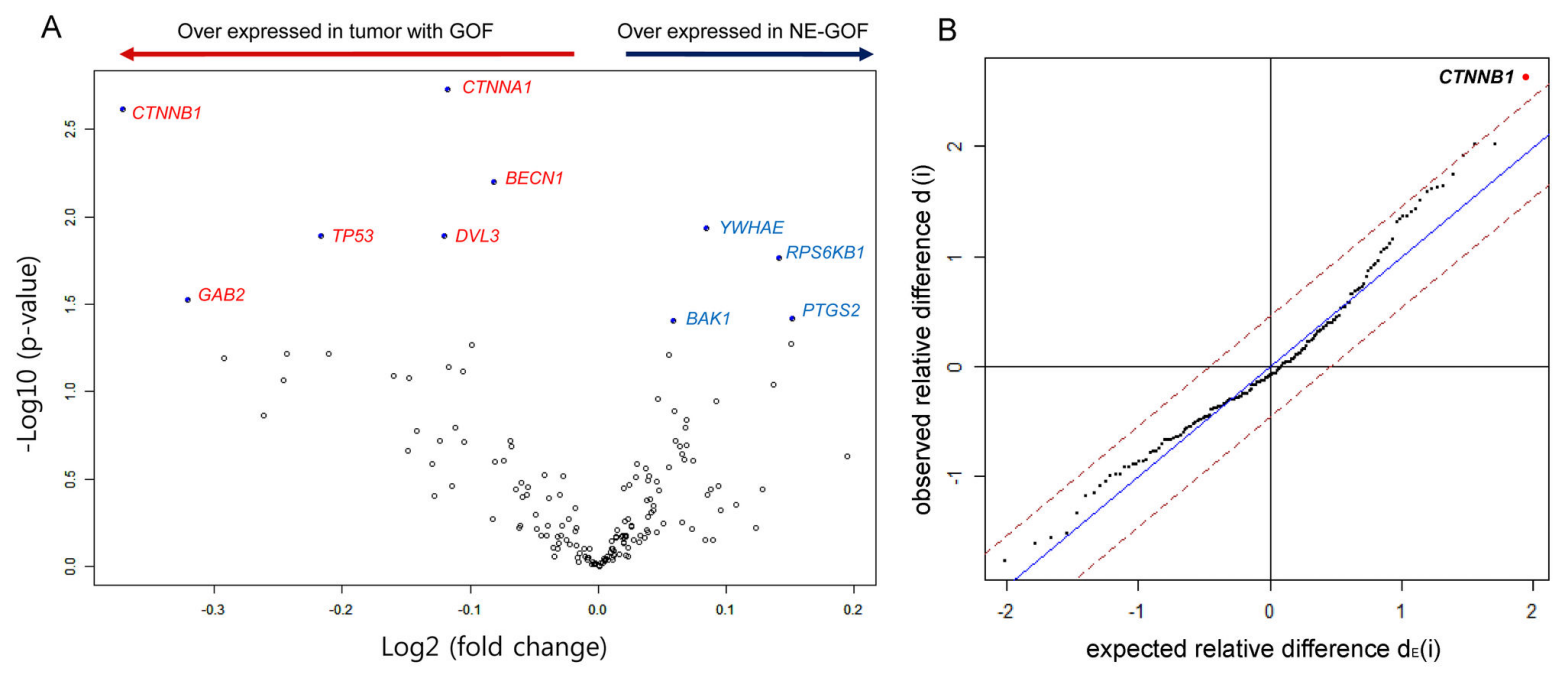
Differential protein expression between gain-of-function (GOF) and no evidence of GOF (NE-GOF) mutations of p53. Among 165 proteins, 11 significant differentially expressed proteins (*p*<0.05) and their fold changes were shown in Volcano plot (A). By the significance analysis of microarrays (SAM) method, only *CTNNB1* was identified as a significant differentially expressed protein between GOF and NE-GOF mutant p53 proteins (B).

Among the 165 proteins in the protein expression array, 10 proteins were found to be differentially expressed between patients with GOF and NE-GOF mutations (parametric *p*-value < 0.05) (Table 2 and Figure 4A). CTNNA1 and CTNNB1 were the most differentially expressed proteins (*p*<0.01 and FDR<0.25). The remaining eight proteins were BECN1, YWHAE, DVL3, TP53, RPS6KB1, GAB2, PTGS2, and BAK1. Using the SAM method, CTNNB1 was identified as a significant protein between the two groups (Figure 4B).

**Table 2:** Differentially expressed proteins in mutant p53 protein with gain-of-function.

**Protein**	**Parametric p-value**	**Permutation p-value**	**FDR**	**Geom mean of intensities in NE of GOF class**	**Geom mean of intensities in GOF class**	**Related function and pathways**
CTN1	0.0019	0.0011	0.201	1.01	1.1	Cell to Cell Adhesion Signaling, Adherens junction, Leukocyte transendothelial migration, Tight junction
CTNNB1	0.0024	0.0029	0.201	4.1	5.3	Cell to Cell Adhesion Signaling, WNT Signaling Pathway, Adherens junction, Focal adhesion, Leukocyte transendothelial migration, Tight junction
BECN1	0.0063	0.006	0.349	1.49	1.58	Regulation of autophagy
YWHAE	0.0116	0.0106	0.356	0.46	0.43	Cell cycle
DVL3	0.0128	0.0131	0.356	0.68	0.74	Notch signaling pathway, Wnt signaling pathway
TP53	0.0129	0.0116	0.356	0.17	0.2	Apoptotic signaling in response to DNA damage, ATM signaling Pathway, Cell cycle, p53 signaling Pathway, Chaperones modulate interferon Signaling Pathway, Role of BRCA1, BRCA2 and ATR in Cancer Susceptibility, Telomeres, Telomerase, Cellular Aging, and Immortality, Tumor Suppressor Arf Inhibits Ribosomal Biogenesis,MAPK signaling Pathway
RPS6KB1	0.0171	0.0166	0.402	1.57	1.43	IL 4 signaling pathway, MAPKinase Signaling Pathway, mTOR Signaling Pathway, Rac 1 cell motility signaling pathway, Regulation of eIF4e and p70 S6 Kinase, Insulin signaling pathway, mTOR signaling pathway, TGF-beta signaling pathway
GAB2	0.0298	0.0278	0.615	1.9	2.38	Fc epsilon RI signaling pathway
PTGS2	0.0381	0.0332	0.651	1.44	1.3	Eicosanoid Metabolism, Mechanism of Gene Regulation by Peroxisome Proliferators via PPARa(alpha), Arachidonic acid metabolism
BAK1	0.0395	0.0374	0.651	1.38	1.33	Role of Mitochondria in Apoptotic Signaling

NE-GOF, no evidence of gain-of-function

## Discussion

In *TP53* mutation, density of mutations and hotspot mutations are associated with DNA binding domain. This study revealed that hotspot mutations are frequently occurred at CpG sites in DNA binding domain and hotspot mutations, functional impairment of mutp53, and GOF mutations are all related. Mutations within hotspots severely impaired the function of p53, which suggests that the occurrence of hotspot mutations is also associated with clonal selection for cancer development. In addition, this study demonstrated that GOF mutations in p53 are associated with hotspot mutations, mutations at CpG sites, and high FS scores.

The clinical relevance of GOF mutations in p53 has never been evaluated systematically in large clinical samples. In this study, HGS-OvCa with GOF mutp53 had a greater metastatic potential and tended to be resistant to platinum-based chemotherapy. It is known that overexpression of various tumor-associated mutp53 can render cells markedly more resistant to killing by a variety of anticancer agents [4]. In addition, resistance to etoposide and cisplatin was observed in human H1299 lung cancer cells overexpressing different types of tumor-associated human mutp53 proteins, notably p53R175H and p53R273H [23]. R175H and R273H are considered to be GOF mutations. Knock-in of p53 R270H or R172H led to a markedly increased incidence of highly metastatic carcinomas in various mouse models [5–10]. Furthermore, whereas knock-down of endogenous mutp53 in MDA-MB-231 human breast cancer cells did not affect primary tumor growth, it strongly reduced metastasis to both the lymph nodes and the lung [24]. This study demonstrated the metastatic and chemoresistant properties of HGS-OvCa tumors with GOF mutp53.

In this study, there was no difference in survival between GOF mutp53 and NE-GOF mutp53, although GOF mutp53 is likely to be associated with platinum treatment resistance and increased metastatic behavior. However, recently, Lang et al. [6] revealed similar results that p53^+/515A^ mice corresponding to the p53R175H hot spot mutation in human cancers showed a similar survival curve with p53^+/-^ mice, although tumors from p53^+/515A^ mice metastasized with high frequency. These findings may suggest that more complex mechanisms are involved in cancer progression and survival.

In this study, we identified several up-regulated proteins, including CTNNA1, CTNNB1, BECN1, DVL3, TP53, and GAB2, and several down-regulated proteins, including YWHAE, RPS6KB1, PTGS2, and BAK1 in patients with GOF mutp53, compared to patients with NE-GOF mutp53. Among them, CTNNB1 (β-catenin) was also identified by the SMA method as being significantly overexpressed in patients with GOF mutp53. Metastasis is associated with epithelial-mesenchymal transition (EMT), resulting in loss of cell-cell adhesion and an increase in cell motility. Mutp53 have been found to promote EMT by facilitating EMT-related key transcriptional factors, such as Twist1 and slug, and by promoting TGF-β-induced metastasis [10,25,26]. CTNNA1 and CTNNB1, which were the most highly differentially expressed proteins in cancers with GOF mutp53 compared to NE-GOF mutp53, are involved in cell-cell adhesion signaling and the WNT signaling pathway, which suggests that the β-catenin signaling pathway, including CTNNB1, is involved in increasing the metastatic properties of tumors with GOF mutp53.

In this study, higher p53 mRNA and protein expressions were present in patients with missense mutation. It is known that mutp53 is stabilized in cancer and escape from E3 ubiquitin ligase-mediated degradation is an important mechanism for mutp53 stabilization. In this study, expression level of MDM2 mRNA was not significantly different between GOF and NE-GOF (MDM2 protein level was not available). Therefore, higher expression levels of p53 mRNA and protein in GOF mutp53 may suggest presence of additional mechanisms that lead to mutp53 stabilization and resulted in more GOF effects. In this study, CTNNB1 (beta-catenin) was a significant differentially expressed protein between GOF and NE-GOF, which may suggest that beta-catenin related pathway is involved in GOF effects and mutp53 stabilization. Recently, Li et al. [27] reported that PTEN can have tumor-promoting properties in cells that harbor GOF p53 mutations and PTEN increased that levels of mutp53 protein by inhibiting its degradation possibly via inhibition of PI3K/Mdm2 and physical binding. PTEN also plays an important role in its ability to regulate beta-catenin through the Wnt Pathway [28].

In this study, classification of GOF mutp53 was based on experiments of published studies [20]. Therefore there may be a limitation in the fact that mutants that more frequently identified in cancer tend to be more studied for functional properties and those mutants are more likely to exhibit GOF properties.

The standard treatment for HGS-OvCa is aggressive cytoreductive surgery followed by platinum-based multi-agent chemotherapy. HGS-OvCa is usually platinum-sensitive [14,29,30]; however, approximately 30% of patients exhibit platinum resistance and particularly aggressive disease progression [17,18,31,32]. Primary resistance to chemotherapy is a major cause of treatment failure. Currently, it is difficult to predict which patients will respond to platinum-based chemotherapy. In addition, it is not known which patients will recur with distant metastasis. This study provides some clues to these issues.

Growing evidence has suggested an oncogenic role for GOF p53 in tumorigenesis, cancer invasion, and metastasis. However, the role of GOF mutp53 had not demonstrated in clinical samples. Although there is no consensus on the molecular definition of GOF mutation, several mutp53 have been demonstrated to have GOF activity. Based on current evidence with known GOF mutations of p53, we demonstrated that GOF mutp53 plays a clinically significant role in patients with HGS-OvCa by conferring platinum resistance and metastatic properties.
